# Synchronous and histologically heterogeneous multifocal neoplasia detected by confocal laser endomicroscopy in gastric squamous ectopia

**DOI:** 10.1055/a-2748-1330

**Published:** 2025-12-17

**Authors:** Shan Wu, Jun Zhou, Xinjian Wan, Zhixia Dong

**Affiliations:** 1 Digestive Endoscopic Center, Shanghai Sixth People’s Hospital Affiliated to Shanghai Jiaotong University School of Medicine, Shanghai, China; 2 Pathology Department, Shanghai Sixth People’s Hospital Affiliated to Shanghai Jiaotong University School of Medicine, Shanghai, China

Gastric squamous epithelial ectopia (GSEE) is rare; however, synchronous, multifocal, and histogenetically distinct early tumors in this setting are even more uncommon. We present an early neoplasia case, highlighting the unique value of confocal laser endomicroscopy (CLE) for the real-time in vivo diagnosis of such complex, multifocal lesions ( Video 1 ).

Synchronous and histologically heterogeneous multifocal neoplasia detected by confocal laser endomicroscopy in gastric squamous ectopia.Video 1

A 68-year-old man underwent esophagogastroduodenoscopy (EGD) due to elevated carcinoembryonic antigen. The EGD revealed a patch of GSEE on the posterior wall of the gastric body. It was identified by a whitish, slightly elevated appearance with a well-demarcated border, approximately 3 cm below the gastroesophageal junction (EGJ; Fig. 1 **a, b** ). At the gastric squamocolumnar junction (SCJ), a 0.6 cm × 0.8 cm reddish, depressed lesion was observed. ME-NBI showed dark brown discoloration with irregular MV/MS patterns ( Fig. 2 **a, b** ). CLE examination revealed a distorted glandular architecture, cellular atypia, and irregular vessels ( Fig. 2 **c, d** ). Additionally, the two other lesions were identified. At the gastroesophageal junction (GEJ), approximately 1 cm above the dentate line, a 2.0 cm × 1.5 cm type 0-IIb lesion with patchy redness was observed ( Fig. 3 **a** ). ME-NBI demonstrated brownish discoloration with regular vessels (JES B1 pattern; Fig. 3 **b** ). CLE showed a white feathery material, exfoliated cells, focal squamous epithelial loss, and dilated intrapapillary capillary loops (IPCLs; Fig. 3 **c, d** ). Adjacent to this, a 0.5 cm 0-IIb yellowish lesion on the gastric mucosa exhibited dark brown discoloration with irregular microvasculature on ME-NBI ( Fig. 3 **e, f** ). Endoscopic submucosal dissection was successfully performed to resect all lesions.

**Fig. 1:**
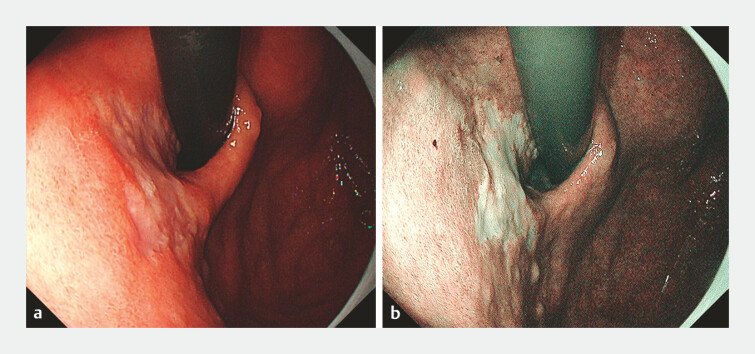
**a, b** Whitish, slightly elevated appearance with a well-demarcated border, approximately 3 cm below the gastroesophageal junction (EGJ).

**Fig. 2:**
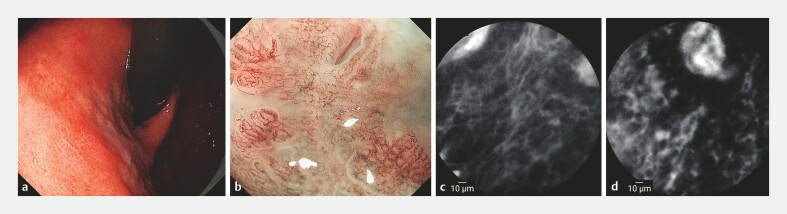
**a, b** At the gastric squamocolumnar junction (SCJ), a 0.6 cm × 0.8 cm reddish, depressed lesion was observed. ME-NBI showed dark brown discoloration with irregular MV/MS patterns. **c, d** CLE examination revealed a distorted glandular architecture, cellular atypia, and irregular vessels.

**Fig. 3:**
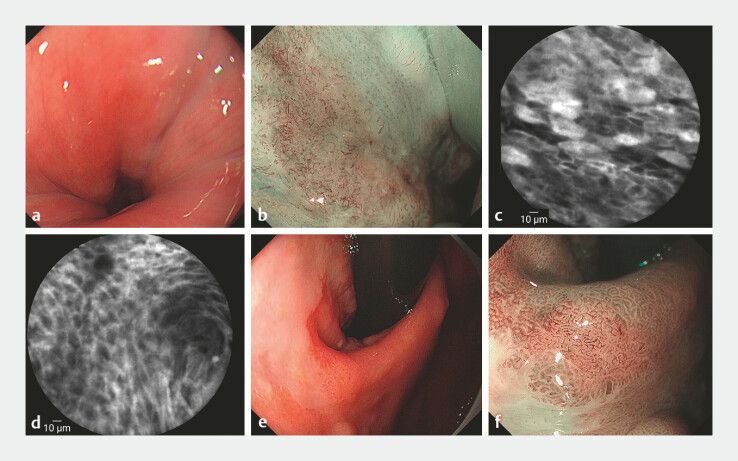
**a** At the gastroesophageal junction (GEJ), a 2.0 cm × 1.5 cm type 0-IIb lesion with patchy redness. **b** ME-NBI demonstrated brownish discoloration with regular vessels (JES B1 pattern). **c, d** CLE showed a white feathery material, exfoliated cells, focal squamous epithelial loss, and dilated intrapapillary capillary loops (IPCLs). **e, f** A 0.5 cm 0-IIb yellowish lesion on the gastric mucosa exhibited dark brown discoloration with irregular microvasculature on ME-NBI.

Histopathological examination revealed the SCJ lesion: moderately–poorly differentiated adenocarcinoma, invading 1600 µm into submucosa with sub-squamous extension ( Fig. 4 **a** ), EGJ lesion: high-grade squamous dysplasia with focal glandular involvement ( Fig. 4 **b** ), and cardiac lesion: superficial low-grade dysplasia with cystic gastritis profunda ( Fig. 4 **c** ).

**Fig. 4:**
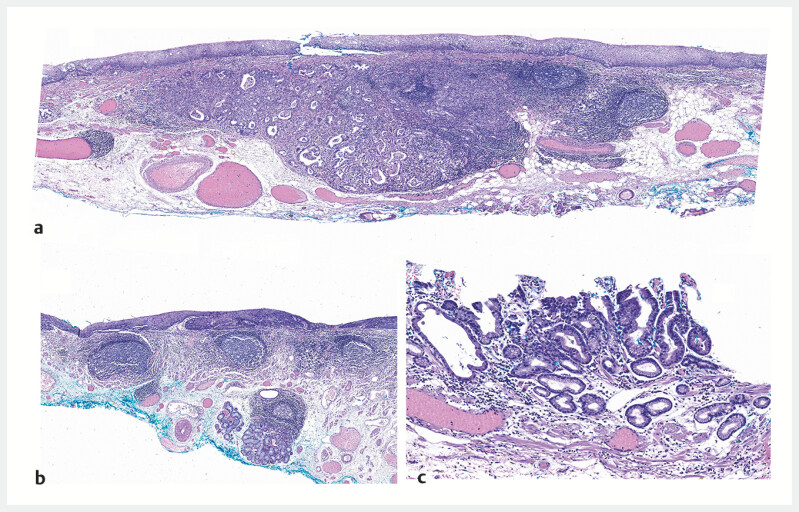
**a** Moderately–poorly differentiated adenocarcinoma (0.6 cm × 0.4 cm × 0.3 cm), invading 1600 µm into submucosa with sub-squamous extension. **b** High-grade squamous dysplasia (3.0 cm × 1.7 cm) with focal glandular involvement. **c** Cystic gastritis profunda with superficial low-grade dysplasia.

CLE demonstrates high sensitivity and specificity in the immediate detection of differentiation of lesions with varying pathological types, serving as a powerful tool in the management of such complex cases.

Endoscopy_UCTN_Code_CCL_1AB_2AD_3AB

## Notes

Endoscopy E-Videos https://eref.thieme.de/e-videos
*E-Videos* is an open access online section of the journal *Endoscopy* , reporting on interesting cases and new techniques in gastroenterological endoscopy. All papers include a high-quality video and are published with a Creative Commons CC-BY license. Endoscopy E-Videos qualify for HINARI discounts and waivers and eligibility is automatically checked during the submission process. We grant 100% waivers to articles whose corresponding authors are based in Group A countries and 50% waivers to those who are based in Group B countries as classified by Research4Life (see: https://www.research4life.org/access/eligibility/ ). This section has its own submission website at https://mc.manuscriptcentral.com/e-videos .

